# Iron scavenging and suppression of collagen cross-linking underlie antifibrotic effects of carnosine in the heart with obesity

**DOI:** 10.3389/fphar.2023.1275388

**Published:** 2024-01-03

**Authors:** Islam A. Berdaweel, T. Blake Monroe, Amany A. Alowaisi, Jolonda C. Mahoney, I-Chau Liang, Kaitlyn A. Berns, Dylan Gao, Jared M. McLendon, Ethan J. Anderson

**Affiliations:** ^1^ Department of Pharmaceutical Sciences and Experimental Therapeutics, College of Pharmacy, University of Iowa, Iowa City, IA, United States; ^2^ Department of Clinical Pharmacy, College of Pharmacy, Yarmouk University, Irbid, Jordan; ^3^ Department of Internal Medicine, Carver College of Medicine, University of Iowa, Iowa City, IA, United States; ^4^ Abboud Cardiovascular Research Center, Carver College of Medicine, University of Iowa, Iowa City, IA, United States; ^5^ Fraternal Order of Eagles Diabetes Research Center, Carver College of Medicine, University of Iowa, Iowa City, IA, United States

**Keywords:** obesity, cardiac fibrosis, lipid peroxidation, carnosine, iron chelation, carbonyl stress, collagen, extracellular matrix

## Abstract

Oral consumption of histidyl dipeptides such as l-carnosine has been suggested to promote cardiometabolic health, although therapeutic mechanisms remain incompletely understood. We recently reported that oral consumption of a carnosine analog suppressed markers of fibrosis in liver of obese mice, but whether antifibrotic effects of carnosine extend to the heart is not known, nor are the mechanisms by which carnosine is acting. Here, we investigated whether oral carnosine was able to mitigate the adverse cardiac remodeling associated with diet induced obesity in a mouse model of enhanced lipid peroxidation (i.e., glutathione peroxidase 4 deficient mice, GPx4^+/−^), a model which mimics many of the pathophysiological aspects of metabolic syndrome and T2 diabetes in humans. Wild-type (WT) and GPx4^+/−^male mice were randomly fed a standard (CNTL) or high fat high sucrose diet (HFHS) for 16 weeks. Seven weeks after starting the diet, a subset of the HFHS mice received carnosine (80 mM) in their drinking water for duration of the study. Carnosine treatment led to a moderate improvement in glycemic control in WT and GPx4^+/−^mice on HFHS diet, although insulin sensitivity was not significantly affected. Interestingly, while our transcriptomic analysis revealed that carnosine therapy had only modest impact on global gene expression in the heart, carnosine substantially upregulated cardiac GPx4 expression in both WT and GPx4^+/−^mice on HFHS diet. Carnosine also significantly reduced protein carbonyls and iron levels in myocardial tissue from both genotypes on HFHS diet. Importantly, we observed a robust antifibrotic effect of carnosine therapy in hearts from mice on HFHS diet*,* which further *in vitro* experiments suggest is due to carnosine’s ability to suppress collagen-cross-linking. Collectively, this study reveals antifibrotic potential of carnosine in the heart with obesity and illustrates key mechanisms by which it may be acting.

## 1 Introduction

Obesity and overweight are chronic metabolic disorders characterized by excessive body fat accumulation with a BMI >30 and 25 kg/m^2^, respectively. Both conditions have approached epidemic proportions globally and are established risk factors for cardiometabolic disorders such as type 2 diabetes mellitus and nonalcoholic fatty liver disease (Piché et al., 2020). “Obesity cardiomyopathy” is a unique clinical condition characterized by cardiac structural remodeling and functional abnormalities independent of any cardiovascular risk factor (e.g., hypertension, coronary artery disease) (Chockalingam, 2022) that is associated with increased risk of premature death (Adams et al., 2006).

Western dietary patterns rich in saturated fat and sucrose are strongly obesogenic and known to cause glucose intolerance, insulin resistance and associated cardiometabolic abnormalities in rodent models (Kopp, 2019). Moreover, this type of diet induces significant lipid peroxidation and protein glycation in cardiovascular tissues (Hauck et al., 2019; Świątkiewicz et al., 2023). Lipid peroxidation is an oxidative reaction between a polyunsaturated fatty acid (PUFA) and a transition metal, usually iron, which is required for its initiation and propagation. If not neutralized by antioxidants, lipid peroxides ultimately degrade into a wide variety of biogenic aldehydes (e.g., 4-hydroxynonenal, HNE, malondialdehyde, MDA) and due to their high reactivity and toxicity these reactive carbonyl species (RCS) have been implicated in the pathology of many obesity related disorders (Davì et al., 2005; Ramana et al., 2013; de Souza Bastos et al., 2016). Pathogenicity of RCS is mainly related to their irreversible modification of proteins and other macromolecules which ultimately lead to changes in cellular metabolism and signaling pathways (Marisa et al., 2012). Lipid peroxidation and its associated RCS are known to be involved in profibrotic signaling through stimulation of collagen production and activation of key mediators such as transforming growth factor β (TGF-β) and other inflammatory chemo-/cytokines (Poli and Schaur, 2000; Macdonald et al., 2001; Albano et al., 2005; Tsubouchi et al., 2019).

As the only member of the glutathione peroxidase superfamily capable of neutralizing lipid peroxides, glutathione peroxidase-4 (GPx4) is a master regulator of ferroptosis and experimental studies have shown that this selenoenzyme has important pathophysiological roles in many cardiometabolic, neurodegenerative, autoimmune diseases and malignancies (Park et al., 2019; Ursini et al., 2022; Wang et al., 2022; Xu et al., 2022). A large number of genetic epidemiological studies have linked *gpx4* variants and GPx4 content/activity with obesity (Rupérez et al., 2014; Costa-Urrutia et al., 2020) cardiovascular and inflammatory diseases (Polonikov et al., 2012; Berdaweel et al., 2022), and cancer (Méplan et al., 2010).

Histidyl dipeptides such as l-carnosine are endogenous molecules endowed with potent carbonyl-scavenging capacity and exist in abundant quantities in excitatory tissues such as skeletal muscle, brain and heart. Carnosine is also capable of buffering protons and chelating divalent cations such as Ca^2+^, properties that have been implicated as mechanisms of cardioprotection in models of cardiac injury (Zhao et al., 2020; Gonçalves et al., 2021). Oral carnosine has shown therapeutic potential in several clinical and experimental models of cardiometabolic diseases in which RCS are known to have a pathogenic role, including insulin resistance and glucose tolerance (Matthews et al., 2021; Rohit et al., 2023), prevention of diabetes related complications (Menini et al., 2020) and cardiovascular disorders (de Courten et al., 2015; Lombardi and Metra, 2016). Most of the beneficial effects of carnosine have been ascribed to its carbonyl-scavenging properties, although carnosine has limited ability to neutralize reactive oxygen species (ROS) directly. In a recent comprehensive study (Anderson et al., 2018), we reported that a carnosine analog which is resistant to degradation by carnosinase can mitigate insulin resistance and liver steatosis/fibrosis in diet induced obesity, even in mice with enhanced lipid peroxidation due to GPx4 haploinsufficiency (i.e., GPx4^+/−^ mice). What remained unclear is whether the beneficial effects of oral carnosine therapy in obesity also extended to the heart, and the potential mechanisms by which it might be acting. In this study, we investigated the effectiveness of oral carnosine supplementation in mitigating the structural and functional changes in the heart that are known to accompany obesity in both wild-type (WT) and GPx4^+/−^ mice. Our findings reveal carnosine to have potent antifibrotic effects in the heart with obesity and identify mechanisms involved in these effects.

## 2 Results

### 2.1 Impact of HFHS diet and oral carnosine supplementation on cardiometabolic parameters

In a previous study we found significantly lower GPx4 enzyme in myocardial tissue from type 2 diabetes patients, corresponding to greater levels of lipid peroxidation and RCS (i.e., HNE adducts) in the tissue from these patients compared with age-matched normoglycemic patients (Katunga et al., 2015). Here, both WT and GPx4^+/−^ mice were randomly assigned to either standard chow diet (CNTL) or HFHS diet for 16 weeks, with half of the HFHS diet group also receiving l-carnosine supplementation in their drinking water (80 mM). The overall study flow and design is summarized in Supplementary Figure S1. After the HFHS diet, the overall body weight, body fat and lean body mass composition were similar among the obese mice with and without carnosine, independent of genotype as shown in Table 1. However, HFHS diet dramatically increased gonadal body fat content (as a percent of body weight) in GPx4^+/−^ mice compared with WT, yet this increase was fully suppressed with carnosine treatment in both genotypes. Notably, WT obese mice developed higher brown fat percent compared to lean WT mice. On the other hand, among the obese GPx4^+/−^ mice, only the carnosine treated group developed significantly higher brown fat content. Carnosine treatment demonstrated a mild enhancement in glycemic control but not insulin sensitivity (data not shown), in both WT mice. Obese mice maintained a normal cardiac output, ejection fraction and stroke volume regardless of carnosine treatment in both genotypes.

**TABLE 1:** Body composition and cardiometabolic parameters.

Parameter	WT CNTL	WT HFHS	WT HFHS + Carnosine	GPx4^+/−^CNTL	GPx4^+/−^HFHS	GPx4^+/−^HFHS + Carnosine	*p*-*value* ^*^
Final body weight (g)	31.02 ± 0.67	50.86 ± 0.95^a^	50.01 ± 0.9^a^	31.56 ± 0.7	49.93 ± 0.4^b^	49.45 ± 0.6^b^	<0.0001
Body fat %	15.79 ± 3.1	43.02 ± 0.6^a^	43.89 ± 0.9^a^	15.82 ± 2.3	46.96 ± 1.1^b^	43.78 ± 0.7^b^	<0.0001
Lean mass%	64.98 ± 2.3	44.87 ± .09^a^	45.17 ± 0.7^a^	65.03 ± 2.0	43.59 ± 0.8^b^	45.82 ± 0.4^b^	<0.0001
Gonadal fat %	2.18 ± 0.1	2.98 ± 0.1^a^	2.59 ± 0.1^c1^	2.08 ± 0.2	4.17 ± 0.1^b,c2^	2.55 ± 0.1^d^	a = 0.0012
							c1 <0.05
							c2, b, d < 0.0001
Brown fat %	1.04 ± 0.1	1.35 ± 0.1^a^	1.38 ± 0.1^a^	1.00 ± 0.1	1.26 ± 0.2	1.49 ± 0.1^b,d^	a, d < 0.05
							b = 0.0003
Glucose AUC (GTT)	4,891 ± 1,046	24,578 ± 1635^a^	18,210 ± 2015^a,c^	11,331 ± 2445	23,145 ± 2589^b^	17,626 ± 4,498 ^d^	a< 0.0001
							b < 0.0006
							d, c < 0.05
Stroke volume (µL	30.88 ± 1.2	37.47 ± 2.3	41.05 ± 3.0	33.60 ± 2.6	44.27 ± 3^b^	41.32 ± 1.184	b < 0.05
Ejection fraction (%)	0.8301 ± 0.003	0.7757 ± 0.02	0.8251 ± 0.01	0.8343 ± 0.01	0.8339 ± 0.02	0.8418 ± 0.01	-
Cardiac output (µL/min)	22,262 ± 1,074	24,037 ± 1,487	27,531 ± 1716	23,068 ± 973.6	28,668 ± 1,679	29,371 ± 1042^b^	b < 0.05
Left ventricle thickness (mm)	0.7011 ± 0.02	0.6897 ± 0.02	0.7591 ± 0.02	0.7694 ± 0.2	0.7638 ± 0.02	0.7376 ± 0.02	-

Values are mean ± SEM., n = 6–12 per group.^*^ Statistical differences between groups were compared using one-way ANOVA, with *post hoc* Tukey’s tests for multiple comparisons between treatments within each genotype.^a^ *p* < 0.05 vs WT-CNTL.^b^ *p* < 0.05 vs GPx4^+/−^CNTL.^c^ *p* < 0.05 vs WT-HFHS.^d^ *p* < 0.05 vs GPx4^+/−^HFHS., Abbreviations: WT; wild type, CNTL; control diet, HFHS*;* high fat high sucrose diet, GTT; intraperitoneal glucose tolerance test.

### 2.2 Effect of HFHS diet and carnosine on GPx4 expression and RCS in myocardial tissue

**FIGURE 1:**
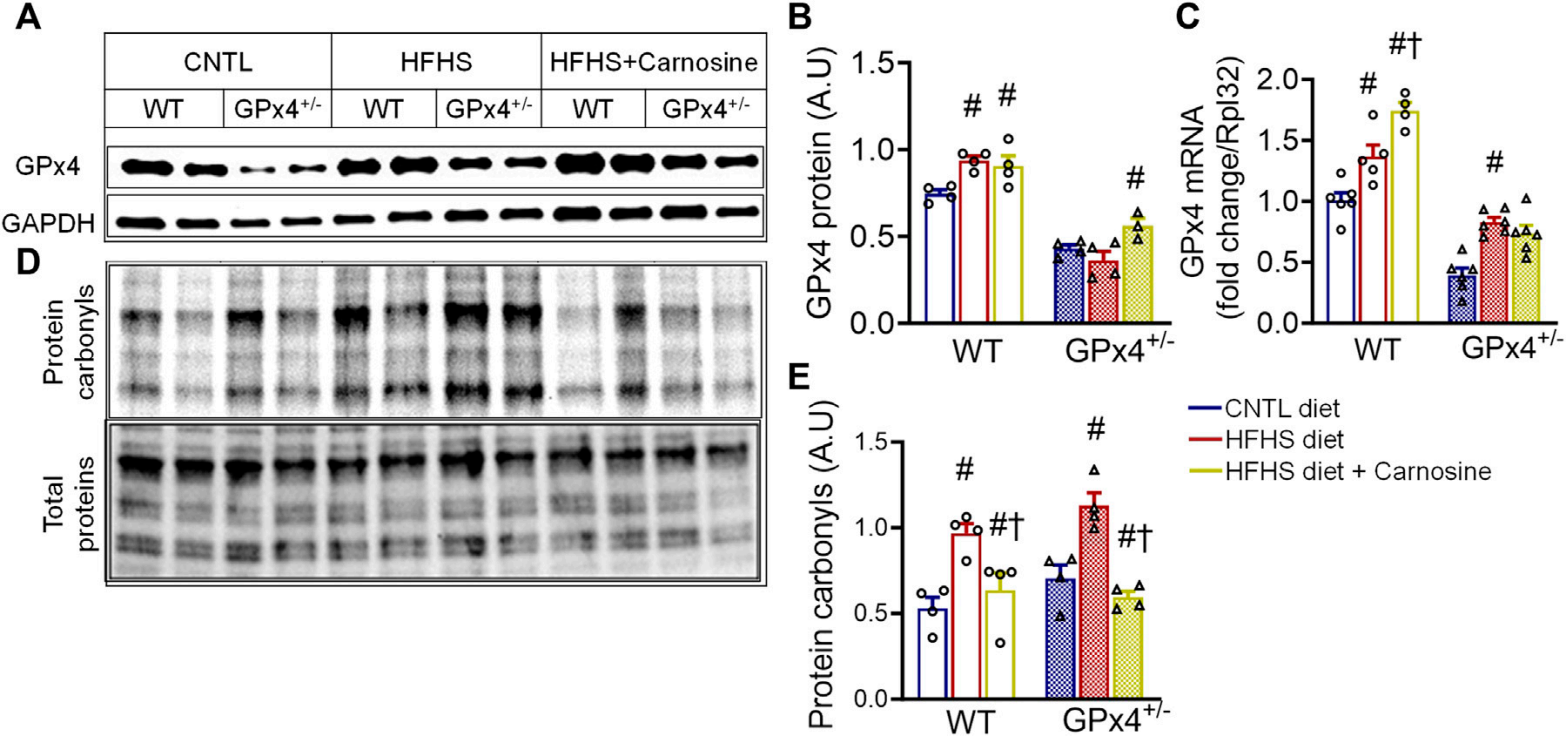
Myocardial GPx4 expression and protein carbonylation. Shown in **(A)** are representative immunoblots of GPx4 protein in left venticular samples with the corresponding densitometric analysis in **(B)**. GPx4 mRNA gene expression is shown in **(C)**. Representative image of Cy5.5 labeled protein carbonyls in whole heart homogenates, n = 2 mice per treatment group is shown in **(D)** with corresponding densitometric analysis shown in **(E)**. One = way ANOVA with *post hoc* Tukey’s tests for multiple comparisons within the same genotype: #*p* < 0.05 vs. CNTL, †*p* < 0.05 vs HFHS. n = 4–6 mice per treatment group. WT, wild type; CNTL, control diet; HFHS, high fat high sucrose diet.

We observed substantial upregulation of myocardial GPx4 mRNA and protein content with HFHS diet and carnosine (Figures 1A–C). To test if this increase in GPx4 was accompanied by other redox enzymes as part of a more global antioxidant response we analysed expression of several other key antioxidant genes and found rather unremarkable changes, with only a small upregulation in glutathione S-transferase (GST) and catalase mRNA with HFHS diet or carnosine (Supplementary Figure S2).

Given that HFHS diet is known to induce lipid peroxidation and RCS formation in the heart, particularly in GPx4^+/−^ mice, (Méndez et al., 2014; Kobi et al., 2023), we next sought to the evaluate the effect of carnosine on protein carbonylation in myocardium from these mice. HFHS diet did indeed increase myocardial protein carbonyls, particularly in GPx4^+/−^ mice, while carnosine treatment effectively blunted protein carbonylation in both genotypes (Figures 1D,E). Since carbonyl stress has been linked with inflammation and fibrosis in many studies (Tanase et al., 2016; Maruyama and Imanaka-Yoshida, 2022), we examined expression of several pro-inflammatory and pro-fibrotic genes and observed a small but significant increase in TGF-β and RAGE expression in the obese GPx4^+/−^ mice as we have previously shown (Katunga et al., 2015), and this upregulation was fully abolished with carnosine treatment. We also observed a significant reduction in iNOS gene expression in the carnosine treated obese mice (Supplementary Figure S3). No significant changes in expression of IL-6, IL-1β and TNF-α were found (data not shown).

We next investigated whether HFHS diet and/or carnosine influenced protein carbonyl levels in the circulation. To do this, we measured serum 4-HNE-protein adducts after food restriction and ∼30 min following glucose challenge. No significant differences in serum 4-HNE protein adducts were found between groups in the fasted (i.e., food restricted) state, but adducts were substantially increased after glucose challenge with all groups, although the greatest increase appeared with HFHS diet. Carnosine treatment blunted the increase in 4-HNE adducts following glucose challenge (Supplementary Figure S4). No differences in overall 4-HNE adducts were found between WT and GPx4^+/−^ mice in any of the treatment groups (data not shown).

### 2.3 Myocardial iron levels following HFHS diet and carnosine

**FIGURE 2:**
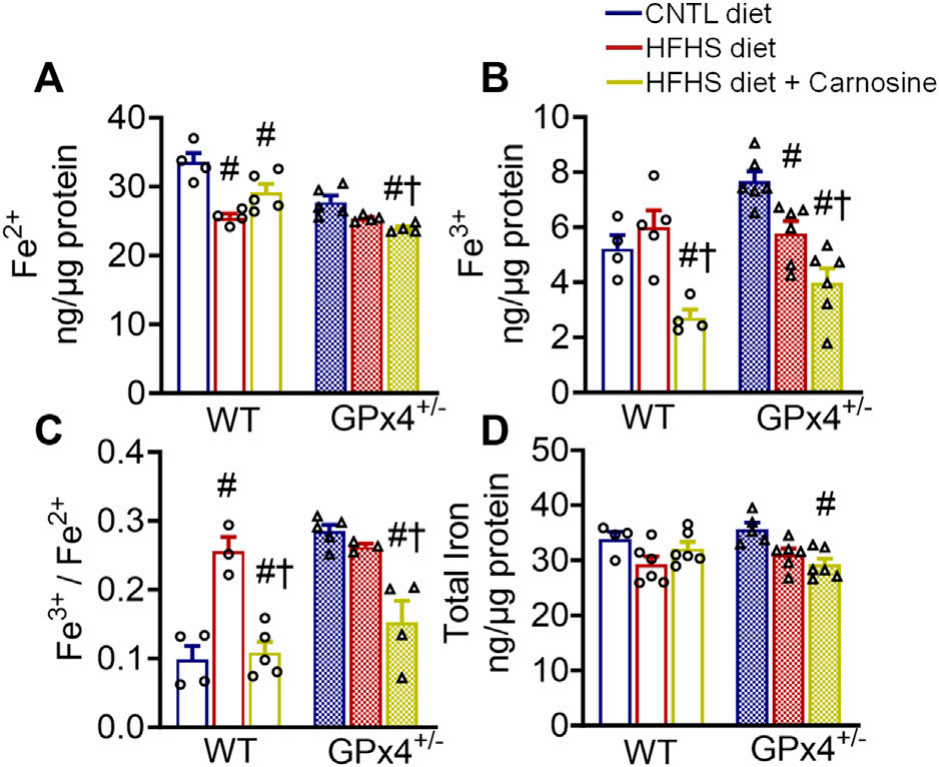
Myocardial iron content. Concentration of ferrous **(A)**, ferric **(B)**, and ferric/ferrous iron ratio **(C)**, in left venticular tissue samples are shown, along with total iron **(D)**. Values represent mean ± SEM. One-way ANOVA with *post hoc* Turkey’s tests for multiple comparisons within the same genotype: #*p* < 0.05 vs. CNTL, †*p* < 0.05 vs. HFHS. n = 4–6 mice per treatment group. WT, wild type; CNTL, control diet; HFHS, high fat high sucrose diet.

Carnosine treatment has been shown to have modest antioxidant effects in several *in vitro* and *in vivo* models, and this effect has been linked partly to its metal chelation ability (Reddy et al., 2005; Hider et al., 2021). Considering the role of iron in the initiation and the propagation of lipid peroxidation (Chen et al., 2021), we next examined myocardial iron levels in the mice from each treatment group. Interestingly, carnosine treatment moderately decreased Fe^2+^ and substantially decreased Fe^3+^ levels in both WT and GPx4^+/−^ mice (Figures 2A–C). Although no significant differences in total myocardial iron levels were found between WT and GPx4^+/−^ mice, carnosine significantly decreased Fe^3+^/Fe^2+^ in HFHS diet in both genotypes (Figures 2C,D). To determine whether this effect on the Fe^3+^/Fe^2+^ ratio is mediated by changes in iron transporters or other regulators, we analyzed expression of several iron homeostasis proteins and found that transferrin receptor 1 (TfR1) expression is increased by carnosine treatment in GPx4^+/−^ mice but not WT. No significant differences in ferritin heavy chain 1 and ferritin light chain were observed, however (Supplementary Figure S5).

### 2.4 Effect of carnosine on cardiac fibrosis and collagen cross-linking with HFHS diet

**FIGURE 3:**
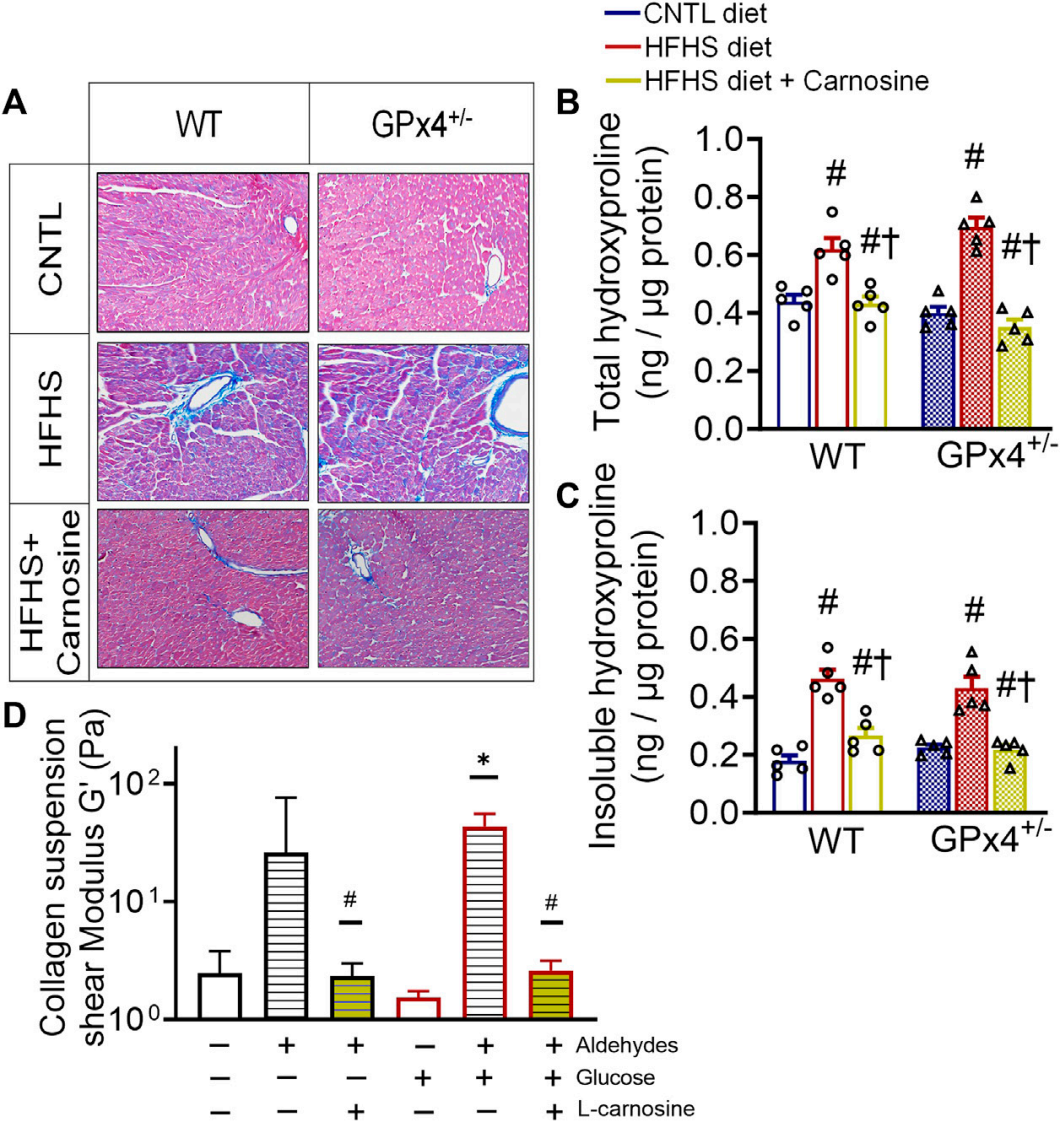
Myocardial collagen, fibrosis and *in vitro* collagen cross-linking. Representative images of Masson’s trichrome staining in fixed myocardial tissue slices shown in **(A)**, n = 2 mice per treatment group, images are representative of 20 image fields captured per mouse under ×20 magnification. Total hydroxyproline concentration **(B)** and insoluble hydroxyproline concentration **(C)** in left ventricular tissue samples, n = 5, values are mean ± SEM, oneway ANOVA with *post hoc* Tukey’s tests for multiple comparisons within the same genotype: #*p* < 0.05 vs. CNTL, †*p* < 0.05 vs HFHS. n = 5 mice per treatment group. Shown in **(D)** is effect of carnosine on collagen cross-linking stimulated by aldehydes and glucose *in vitro* as outlined in the Methods section. Suspensions of collagen were treated with a mixture of biogenic aldehydes (HNE 50 M, DOPAL 10 M, DOPEGAL 10 M) alone or with L-carnosine (10 mM). Elastic moduli of collagen were measured by rheometer at w = 2.122 Hz *p < 0.05 *versus* untreated control, #*p* < 0.05 *versus* aldehyde-treated group for each respective ± glucose. WT, wild type; CNTL, control diet; HFHS, high fat high sucrose diet; GTT, oral glucose tolerance test.

Many previous studies have shown that obesity is accompanied by extracellular matrix protein expansion as a result of collagen deposition in the heart (Tikellis et al., 2008; Natarajan et al., 2019). Histological staining of fixed myocardial tissue with Masson’s trichrome confirmed that fibrosis was mitigated by carnosine in both WT and GPx4^+/−^ mice with HFHS diet (Figure 3A). Quantitative analysis of myocardial hydroxyproline, a marker for collagen, was significantly higher in both WT and GPx4^+/−^ mice with HFHS diet when compared to lean mice, and carnosine treatment completely normalized these levels (Figure 3B). Furthermore, concentrations of the insoluble form of hydroxyproline, which indicates degree of collagen cross-linking in the tissue, was also normalized by carnosine treatment (Figure 3C).

To gain mechanistic insights on the antifibrotic mechanisms of carnosine therapy, we took advantage of the rheological properties of collagen suspensions, namely, its elastic moduli, and how elasticity changes occur when collagen becomes ‘stiff’ in response to cross-linking. Here, using a modified protocol previously established (Aslanides et al., 2016) we performed an *in vitro* study with soluble collagen to determine the effect of a mixture of reactive biogenic aldehydes and glucose on collagen cross-linking using rheometry. The aldehyde mixture included 4-HNE (lipid-derived aldehyde), 3,4-dihydroxyphenylacetaldehyde (DOPAL) and 3,4-dihydroxyphenylglycolaldehyde (DOPEGAL). The latter are dopamine and norepinephrine metabolites, respectively, which we have previously shown are biogenic aldehydes formed in heart and are increased in myocardial tissues from patients with T2 diabetes (Monroe and Anderson, 2021; Nelson et al., 2021). We found that collagen cross-linking was stimulated by these aldehydes in the absence (*p* = 0.0545) and presence of glucose, as indicated by higher shear modulus of the suspension (Figure 3D). However, addition of carnosine to the mixture completely suppressed the aldehyde induced cross-linking.

### 2.5 Effect of carnosine on global cardiac gene expression with HFHS diet

**FIGURE 4:**
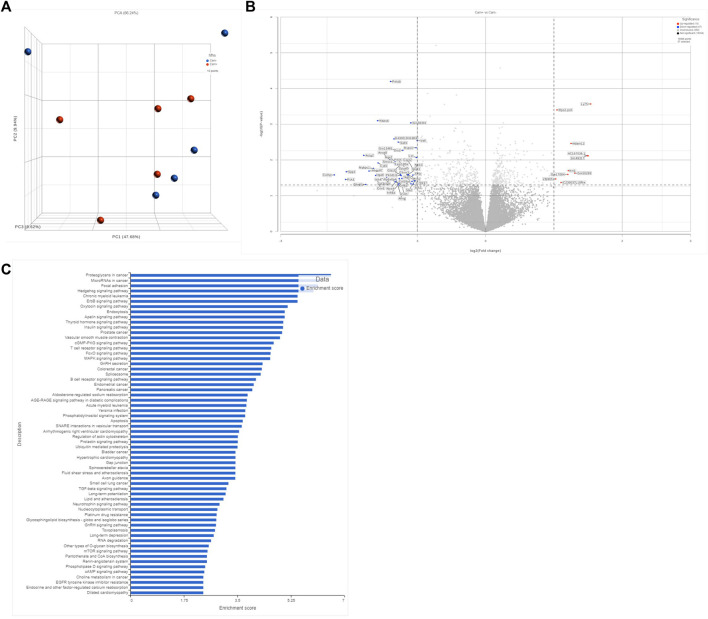
Transcriptomic anlysis of bulk myocardial tissue. Comparison of unbiased transcript expression in myocardial tissues from WT mice on HFHS diet with and without carnosine treatment. N = 5 per treatment group. Shown in **(A)** is a PCA plot accessing sample variance among group. Shown in **(B)** is a volcano plot depicting the top 250 differentially expressed sp transcripts, with the significant upregulated and downregulated genes annotated. *Y*-axis represents significance as −Log10 (*p*-value). *X*-axis represents the Beta value (natural log of the fold change between groups). Shown in **(C)** is a gene ontology pathway analysis based on the filtered list of genes with minimum *p*-value of 0.1 and minimum read count of 50 in at least 50 samples.

To further interrogate potential mechanisms of carnosine’s antifibrotic effects, we performed unbiased mRNA sequencing analysis on myocardial tissue from mice in each experimental group. As expected, major changes in gene expression were found in hearts between WT and GPx4^+/−^ mice (not shown) and between CNTL and HFHS diet (not shown). Interestingly, carnosine only influenced a modest amount of overall gene expression in the hearts when compared with HFHS diet alone, with <50 transcripts passing the threshold of significance in our analysis for either up- or downregulation (Figures 4A,B). Of note, KEGG pathway analysis of differentially expressed gene pathways did list AGE-RAGE and TGF-β as significantly changing with carnosine (Figure 4C and Supplementary Table S3), which support our qRT-PCR results.

## 3 Discussion

Fibrosis is a significant pathogenic contributor to most cardiac disorders including valve disease, cardiomyopathies, arrhythmias and heart failure. Unfortunately, at present there are essentially no pharmacotherapies used in the clinic that specifically target fibrosis. This is partly due to the complex role of fibrosis in wound healing combined with the fact that the mechanisms controlling extracellular matrix expansion and collagen turnover in heart remain incompletely understood. Moreover, obesity induced cardiac fibrosis is a complex heterogenous condition involving a wide variety of molecular pathways. Efforts to understand fibrosis and its mechanisms could be of great benefit for developing new or existing therapies for cardiac fibrosis that could help lower incidence of cardiovascular diseases and their global public health burden (Wong et al., 2012; Travers et al., 2016; Hinderer and Schenke-Layland, 2019; Travers et al., 2022). To this end, our study demonstrates that oral administration of the histidyl dipeptide L-carnosine mitigates carbonyl stress in the heart with diet induced obesity and this consequently leads to less cross-linked collagen and ultimately, less cardiac fibrosis. Furthermore, the cardioprotective effects of oral carnosine are likely multifactorial and indirect, given that cardiac transcriptomic analysis of carnosine treated mice showed only modest differences compared with untreated animals. Much of the indirect effects of carnosine involve its iron chelating ability and its role as an indirect antioxidant, enhancing GPx4 expression in the heart following HFHS diet even in mice with GPx4 haploinsufficiency.

Carnosine is an endogenous dipeptide that has been extensively investigated as a cardio/neuroprotectant in numerous studies including atherosclerosis (Barski et al., 2013), diabetes and its associated micro and macrovascular complications (Lee et al., 2005; Caruso et al., 2023), aging (Hipkiss et al., 2016) and various neurodegenerative disorders (Caruso et al., 2019; Solana-Manrique et al., 2022). It is widely accepted that carnosine does have some direct antioxidant effect, including ability to neutralize ROS, RCS and reactive nitrogen species (Hipkiss, 2009; Boldyrev et al., 2013; Caruso et al., 2017; Aldini et al., 2021). However, the direct antioxidant effect of carnosine is unlikely to be a primary therapeutic mechanism *in vivo* given the high concentrations of carnosine required for these effects, and accumulating evidence suggests that alternative therapeutic mechanisms are involved. In this study, we found that oral carnosine supplementation induces significant GPx4 expression and content in the heart. Such an effect of carnosine has been shown previously by other groups, where carnosine treatment normalized lipid peroxidation levels and glutathione ratio, increased superoxide dismutase (SOD) and catalase (CAT) activities in rat brain treated with salsolinol (a toxic pesticide) (Zhao et al., 2017). Similarly, carnosine supplementation was found to increase the expression and activities of SOD, CAT, and glutathione peroxidase (GPX) in the liver, muscles, and plasma of pigs (Caruso et al., 2017).

Studies have also shown carnosine’s effectiveness as a chelator of divalent cations, including iron. Iron is a transition metal that exists in either ferric (Fe^3+^) or ferrous (Fe^2+^) forms in biological systems. In our study, carnosine treatment modulated the redox equilibrium between Fe^3+^/Fe^2+^ ions in the myocardium of obese mice (Figure 2), leading to dramatic decrease in the Fe^3+^/Fe^2+^ ratio. Fe^2+^ ions react with hydrogen peroxide to produce Fe^3+^ ions as well as hydroxyl free radicals (OH^●-^) that initiate lipid peroxidation through a Fenton-based mechanism (Minotti and Aust, 1987a; Latunde-Dada, 2017). The classical dogma regarding this reaction has always maintained that Fe^2+^ is the form required to initiate lipoperoxidation. Importantly, it has been shown that the presence of Fe^2+^ alone is not sufficient to induce lipid peroxidation, while the addition of Fe^3+^/Fe^2+^ in 1:1 ratio potently induces lipid peroxidation (Minotti and Aust, 1987b; Braughler et al., 1987). This finding was confirmed by a recent study which showed that addition of Fe^3+^ to the reaction mixture stimulates lipoperoxidation (Ohyashiki et al., 2002). Moreover, the importance of the Fe^3+^/Fe^2+^ ratio under different peroxidation conditions was demonstrated in classic redox equilibria studies which concluded that this ratio directly regulates the rate and extent of lipid peroxidation (Braughler et al., 1986; Minotti and Aust, 1987b; Ohyashiki et al., 2002). Although the absolute ratio of Fe^3+^/Fe^2+^ was variable among these studies depending on the reaction components (e.g., oxidants, lipids), what is undeniably clear is that higher Fe^3+^/Fe^2+^ favors a more rapid and extensive lipid peroxidation (Tadolini and Hakim, 1996; Tadolini et al., 1997). This might be partly explained by the possibility that Fe^3+^ ions could complex with polyunsaturated fatty acids at their ∆^9^ or ∆^11^ carbon constituent, thus facilitating their peroxidation, as shown recently (Morrill et al., 2004). Given the known link between lipid peroxidation and ferroptosis, it is notable that a higher Fe^3+^/Fe^2+^ ratio was found to be associated with ferroptosis in serum of Eales disease patients and in the amyloid plaque regains of mouse brains in an Alzheimers’ model. Both conditions are associated with lipid peroxidation and advanced glycation end product (AGE) accumulation (Swamy-Mruthinti et al., 2002; Selvi et al., 2007; Wu et al., 2023). However, the prooxidant effect of iron in these studies is typically examined in the context of total iron levels rather than reporting the Fe^3+^/Fe^2+^ ratio, which makes the *in vivo* and translational significance of this observation somewhat limited.

In addition to its ability to chelate metals, it is well established that carnosine is a potent RCS scavenger, thereby inhibiting protein carbonyl formation and its consequent effects in cells and tissues. Consistent with this, our findings show that carnosine treatment reduces the overall level of oxidative protein damage in the myocardium, as indicated by reduced protein carbonyls in the tissue (Figure 1). Protein carbonyls are considered a form of irreversible post-translational protein modification and have been found to cause derangements in cellular metabolism and signaling and implicated in the pathogenesis of obesity (Macdonald et al., 2001; Grimsrud et al., 2008; Méndez et al., 2014; Katunga et al., 2015; de Souza Bastos et al., 2016). Products of lipid peroxidation such as the α,β-unsaturated carbonyls (e.g., 4-HNE) react with the nucleophilic residues in proteins (e.g., Cys, Lys, His and Arg) resulting in the formation of inter and intramolecular cross-links (Spickett and Pitt, 2019). Protein carbonylation is also caused by AGEs (i.e., sugar-amines) which are forms of oxidized glucose-derived aldehydes generated under chronic hyperglycemic conditions (Gaar et al., 2020). Structural changes in proteins caused by carbonylation leads to protein unfolding and polymerization, ultimately leading to formation of insoluble cross-linked protein aggregates. These protein aggregates evade proteasomal degradation, which prolongs their turnover rate and enhances their accumulation (Negre-Salvayre et al., 2008; Dilek, 2022).

It is in this context where the major antifibrotic effect of carnosine appears to exist, based on our findings. The extracellular matrix is primarily a collagen-based network (96%) that is highly susceptible to crosslinking on Lys and Arg residues within the network (Maruyama and Imanaka-Yoshida, 2022). Measuring the extent of collagen crosslinking by assessing the concentration of insoluble compared with soluble collagen is routinely used as an index of collagen cross-linking in tissues, and enhanced crosslinking in the heart has been reported in cardiometabolic disorders such as hypertension (Woodiwiss et al., 2001; Badenhorst et al., 2003) and diabetes (Avendano et al., 1999; Liu et al., 2003). Importantly, collagen crosslinking has been recognized as a key modulator of left ventricular diastolic stiffness in patients with heart failure (Asif et al., 2000; Klotz et al., 2005). The anti-carbonyl and anti-crosslinking effect of carnosine was initially reported by Hipkiss et al. (Hipkiss and Brownson, 2000) and others (Carini et al., 2003; Baba et al., 2013; Albrecht et al., 2017), the mechanism of which has mainly been attributed to carnosine’s ability to react with RCS through Michael addition resulting in carnosine-adducts that are detoxified by dehydrogenases (Bispo et al., 2016). *In vitro* studies have shown that carnosine’s anti-crosslinking effect was able to preserve activities of several enzymes such as SOD (Hipkiss et al., 1995), esterase (Yan and Harding, 2005) and aspartate aminotransferase (Swearengin et al., 1999). In the present study, carnosine was able to block *in vitro* collagen crosslinking stimulated by a mixture of biogenic aldehydes and sugars (Figure 3), and this was reflected in the obese mice where carnosine substantially decreased the concentration of insoluble collagen in the heart.

To conclude, our study has revealed that oral carnosine therapy has potent antifibrotic and carbonyl detoxifying effects in the heart with diet induced obesity. Translational significance of these findings remains to be determined and there are challenges with oral carnosine therapy in humans due to serum carnosinase activity (Menini et al., 2012; Vistoli et al., 2012; Boldyrev et al., 2013). However, carnosinase-resistant analogs of carnosine have shown similar beneficial effects in rodent models of obesity and cardiovascular disease (Menini et al., 2012; Anderson et al., 2018). It is noteworthy that multiple small clinical trials of oral carnosine therapy have shown modest but significant cardiometabolic benefits in obese, prediabetic individuals (Côté et al., 2012; de Courten et al., 2016; Baye et al., 2017; Baye et al., 2018). Future work is obviously needed to optimize and exploit therapeutic efficacy of carnosine in humans.

## 4 Materials and methods

### 4.1 Mouse model, diet and oral carnosine intervention

Animal care and experimental procedures were reviewed and approved by the University of Iowa Institutional Animal Care and Use Committee (IACUC) prior to beginning the study. GPx4^*+/−*^ mice were generated as previously described and maintained by backcross with C57BL6/J (Jackson Laboratory) mice. At 8–10 weeks, male GPx4^+/−^ and WT littermates were randomly assigned to either normal chow diet (CNTL, D20122207, Research Diets, Inc) or high fat high sucrose diet (HFHS), D09071704, Research Diets, Inc) for 16 weeks. Seven weeks after starting the diet, a subgroup of the mice on HFHS diet in both genotypes was started on 80 mM carnosine supplemented in their drinking water and the diet intervention was continued. The mice randomization was continued until there were 10 mice per treatment group Diets were matched for protein and macronutrients except that the HFHS diet is comprised of lard-based fat (35.5% daily kcal) and cholesterol (1.5% daily kcal), plus sucrose (38% daily kcal), compared with the CNTL diet with low fat (6% daily kcal), and starch-based carbohydrates (∼75% daily kcal). HFHS diet and water were refreshed every 3–4 days. Carnosine was confirmed to be completely stable for this length of time in drinking water using HPLC (Supplementary Table S1). At the end of the diet −/+ carnosine intervention, the mice were euthanized after 8 h fast followed by either a 1 g/kg bolus (i.p.) of 50% dextrose in saline, or normal saline.

### 4.2 Metabolic and cardiovascular parameters

Body weight was recorded at baseline and then once every week during the diet intervention. An intraperitoneal glucose tolerance test (GTT) was performed 14 weeks after starting the diet using standard technique. Briefly, a dose of 1 g/kg of 50% dextrose in saline was administered by i. p. injection after 6 h of fasting, followed by blood glucose measurements using glucometer (OneTouch, Verio Flex). Body composition was determined in each animal within 24 h of euthaniasia by the *Time Domain NMR Analyzer* (LF50, Beckman Coulter). Echocardiography in mice was performed 1 week prior to euthanasia in conscious mice using a 30 MHz transducer (Vevo 2100, VisualSonics, Toronto, ON), by staff in the cardiovascular phenotyping core facility at University of Iowa.

### 4.3 Masson’s trichrome stain

Mice were anesthetized with ketamine xylazine and perfused via cardiac puncture with 4% paraformaldehyde (PFA) for 20 min. Hearts were then sectioned and stained at the University of Iowa Pathology Core, according to standard procedures. Briefly, paraffin-embedded hearts were sectioned at 5-µm thickness, then deparaffinized and rehydrated with serial dilutions of concentrated alcohol (100% alcohol, 95% alcohol 70% alcohol). Sections were then re-fixed in Bouin’s solution for 15 min at 56°C and rinsed with water for 5–10 min to improve staining quality. Sections were then incubated in Weigert’s iron hematoxylin solution for 10 min then washed in running warm water buffer for 5 min. Then sections were stained with Biebrich scarlet acid fuchsin solution for 10–15 min and washed in distilled water. Sections were then subjected to differentiation in phosphomolybdic-phosphotungstic acid solution for 10–15 min and transferred immediately to aniline blue solution and stained for additional 5–10 min. This last stain was followed by a brief rinse in distilled water and differentiation in 1% acetic acid solution for ∼5 min. Lastly, sections were dehydrated using 95% ethyl alcohol, absolute ethyl alcohol and xylene. Stained sections were visualized by a EVOS FL AUTO2 microscope (ThermoFisher, Inc.) and images captured by camera with ×10 objective.

### 4.4 Immunoblot analysis

Samples of left ventricular tissue (10–15 mg) were homogenized in TEE buffer (10 mM Tris, 1 mM EDTA, 1 mM EGTA, 0.5% Triton X-100 pH 7.4) with a protease/phosphatase inhibitor cocktail (Roche) using a glass grinder (Kimble Chase, Vineland, NJ, United States). Lysates were then mixed with 10% β-mercaptoethanol and Laemmli buffer and loaded onto 4%–20% gradient polyacrylamide gel (Bio-Rad, Hercules, CA) and subjected to electrophoresis. Proteins were then transferred from gel to a PVDF membrane via semi-dry apparatus, blocked in 3% bovine serum albumin, and incubated with primary antibodies for GPx4 and GAPDH (Abcam, Cambridge, UK). Membranes were then incubated with horseradish peroxidase (HRP) conjugated goat anti-rabbit secondary antibody and scanned by the iBright Imaging System (iBright FL1000, ThermoFisher Scientific, Waltham, MA, United States). Densitometric analysis was performed using ImageJ (NIH).

### 4.5 Quantitative analysis of serum 4-HNE adducts

For quantitative analysis of 4-HNE adducts in serum, an enzyme-linked immunosorbent assay (ELISA) established by our group was used (Monroe and Anderson, 2019). In brief, standards of 4-HNE-protein adducts were first made by incubating serial dilutions of 4-HNE (Cayman chemicals, United States) in 1 mg/mL BSA/50 mM sodium phosphate buffer (pH = 7.4) at 37 C° for 24 h. Serum and standards were loaded in duplicate on immunolon-coated 96-well plates (Nunc MaxiSorp, Invitrogen, United States) and incubated overnight at 4C° with a continuous rocking. The plate was then blocked with 10% BSA for 2 h followed by overnight incubation with 4-HNE polyclonal primary antibody (Sigma-Aldrich). Next the plate was incubated with a HRP-conjugated mouse anti-goat secondary antibody followed by incubation with 55 µM Amplex red (fluorogenic reagent) for 30 min at room temperature. Fluorescence intensity of each well was then measured using the BioTek Synergy microplate reader at 530/595 nm (ex/em), concentrations of 4-HNE protein adducts in the serum samples were determined against the standard curve.

### 4.6 Hydrazide labeling of carbonyl-modified proteins in myocardial tissue

For unbiased labeling of protein carbonyls in the myocardial tissues we used a protocol previously established by our group (Katunga et al., 2015). Homogenates of left ventricular tissue (∼10 mg) were prepared using TissueRuptorII (Qiagen) under anaerobic conditions in a nitrogen-saturated glove box (Coy Laboratory Products, Grass Lake, MI) using degassed TEE buffer. Hydrazide Cy5.5 dye (Lumiprobe, Maryland, United States), was incubated with the protein lysates at final concertation of 25uM for 2 hours on an orbital shaker at room temperature, then incubated overnight at 4 C°. Lysates were then loaded into 4%–20% gradient acrylamide gels (Bio-Rad, Hercules, CA) and subjected to electrophoresis. The Cy5.5 label on carbonyls was then captured by imaging the gel with the iBright Imaging System, and total protein was captured using No-Stain protein labeling reagent (ThermoFisher Scientific, Waltham, MA, United States). Images were analyzed by densitometry using ImageJ (NIH).

### 4.7 Myocardial iron content

Iron content of myocardial tissue samples was calculated using a colorimetric iron assay kit (Abcam) according to the manufacturer’s instructions. Briefly, around 15 mg of the heart tissue were lysed using the assay buffer provided with the kit. Standards and samples were loaded into a 96-well plate and incubated with iron reducer at 37 C for 30 min. Next, iron probes were added to the wells and incubated at 37 C for 30 min. Sample absorption was then measured in duplicate at 593 nm with a plate-reader (Epoch, Bio-Tek).

### 4.8 Quantitative analysis of myocardial collagen concentrations

To measure the amount of the soluble and cross-linked collagen separately in the heart samples, we used a protocol for measuring hydroxyproline as a surrogate for collagen using a method established previously by our group (Anderson et al., 2018), which involves first separating the soluble from insoluble (i.e., cross-linked) collagen fractions as described below.

#### 4.8.1 Soluble and insoluble collagen fractionation

Pepsin (Sigma-Aldrich) was dissolved in phosphate-buffered saline (pH 3.0) and then incubated with frozen left ventricular tissue sample (40 μg pepsin/mg tissue) to digest the heart samples at 37C° for 30 min with gentle shaking. The digestion was then stopped by adding 2% SDS, 0.6M β-mercaptoethanol solution to each tissue suspension. This was followed by a 30-min sonication step to enhance the release of the soluble portion into the solution. Soluble protein fraction was then separated from the insoluble fraction by centrifuging at 10000xg for 90 min at 4C°. The soluble fraction (the supernatant) was then separated from the insoluble protein fraction (pellet) into prelabeled glass tubes. The pellets were then resuspended in DDI water, and both sets of samples were completely dried by heating at 100C° overnight. Hydroxyproline concentration in each sample was then quantified by a colorimetric assay as described below.

#### 4.8.2 Hydroxyproline assay

Dried protein within the glass tubes were hydrolyzed using HCl (6 M) at 100C° for 24 h. The HCl was then evaporated by heating at 100C° overnight and samples were resuspended in 50% isopropanol. Next, commercially obtained hydroxyproline (Sigma-Aldrich) was used to prepare standards in DDI water, and then standards and unknown samples were oxidized with chloramine-T solution (1.4%) for 5–7 min at room temperature. The oxidized standards and samples were then mixed with a solution of 4-dimethylaminobenzaldehyde (DMAB) in 60% perchloric acid and incubated for 17 h at room temperature in the dark. After that, absorption of the samples was measured in duplicates at 568 nm using a plate reader (Epoch, Bio-Tek). Concentration of hydroxyproline in the samples was then calculated using the hydroxyproline standard curve within each plate and normalized to the total protein concentration in each sample.

### 4.9 Rheometric collagen cross-linking assay

Collagen suspensions were prepared in phosphate buffered saline (PBS) and were mixed with either 10 µM DPEGAL or 25 mM glucose. Also, the same mixtures were prepared in addition to 10 mM carnosine treatment and incubated for 48 h at 37 C°. The elastic moduli of these collagen suspensions were measured at *ω* = 1.193 Hz using HAAKE™ RheoStress™ 1 Rheometer (Thermo Scientific) that utilizes HAAKE™ RheoWin3 software. The samples were poured between the bottom and the top plates and then a dynamic strain sweep to determine the linear viscoelastic range and the small amplitude oscillatory shear (SOAS) measurements were performed at a frequency ranging between 0.0895 Hz and 15.92 Hz.

### 4.10 RNA extraction and gene expression analysis using quantitative RT-PCR

Total RNA was extracted from left ventricular tissues (∼10 mg), using the RNeasy Fibrous Tissue Kit (QIAGEN, Germantown, Maryland). Reverse transcription was performed with Superscript IV (ThermoFisher Scientific, Waltham, MA, United States) kit according to manufacturer’s instructions. Quantitative PCR (qPCR) was performed according to the manufacturer’s protocol on the ViiA 7 real-time PCR system (Applied Biosystems, ThermoFisher Scientific, Waltham, MA, United States) using the PowerTrack™ SYBR Green Master Mix (ThermoFisher Scientific, Waltham, MA, United States). Primers used for each target are listed in Supplementary Table S2. Amplification curves were established and expression of each target mRNA was calculated using the ΔΔ Ct (threshold cycle) method. Fold-change differences in target mRNAs were expressed relative to WT-CNTL as the baseline.

### 4.11 Transcriptomic profiling of myocardial tissue

mRNA sequencing of total bulk RNA myocardial tissue was performed by the University of Iowa Genomics Division, using commercial protocols provided by vendors. Briefly, 500 ng of total RNA (DNAse-1 treated) was used to prepare sequencing libraries using the Illumina TruSeq stranded mRNA library preparation kit (Cat. #RS-122–2101, Illumina, Inc., San Diego, CA). Final concentrations of the resulting indexed libraries were determined using the Fragment Analyzer (Agilent Technologies, Santa Clara, CA) and pooled equally for sequencing. Library pool concentration was determined using the Illumina Library Quantification Kit (KAPA Biosystems, Wilmington, MA) and sequenced on a SP flowcell of the Illumina NovaSeq 6,000 genome sequencer using 150 bp paired end SBS chemistry, at a read depth of ∼35M reads per sample. RNA sequencing data was aligned using STAR version 020,201 to the GRCm38.98 release. The average mapping rate across samples was ∼90% and read depth was ∼25M aligned reads per sample. BAM files were uploaded to Partek Flow and feature counts performed on ensemble gene annotations. Gene level differential expression was performed using DESeq2 after median ratio normalization. Data visualizations (Volcano, PCA, cluster plots) were created using Partek flow software.

### 4.12 Statistical analysis

All the data in Table 1 are presented as mean ± SEM. Statistical analysis on mouse model variables were performed with GraphPad Prism (GraphPad Prism, La Jolla, Ca.). One-way ANOVA was performed on continuous variables followed by Tukey’s post-tests for multiple comparisons between treatment groups within the same genotype. Statistical significance between groups was defined as *p* < 0.05. For RNA-seq data, the level of significance has been set at *p* < 0.05 and fold change >1 up or down. See Supplementary Table S3.

## Data Availability

The original contributions presented in the study are publicly available. This data can be found here: https://www.ncbi.nlm.nih.gov/geo/query/acc.cgi?acc=GSE250315.
