# Supplementary material for: Iron scavenging and suppression of collagen cross-linking underlie antifibrotic effects of carnosine in the heart with obesity
Source: Front Pharmacol. 2024 Jan 3;14:1275388. doi: 10.3389/fphar.2023.1275388 (PMC10859874; doi:10.3389/fphar.2023.1275388)
Supplement: Supplementary file 1 [file DataSheet1.pdf]

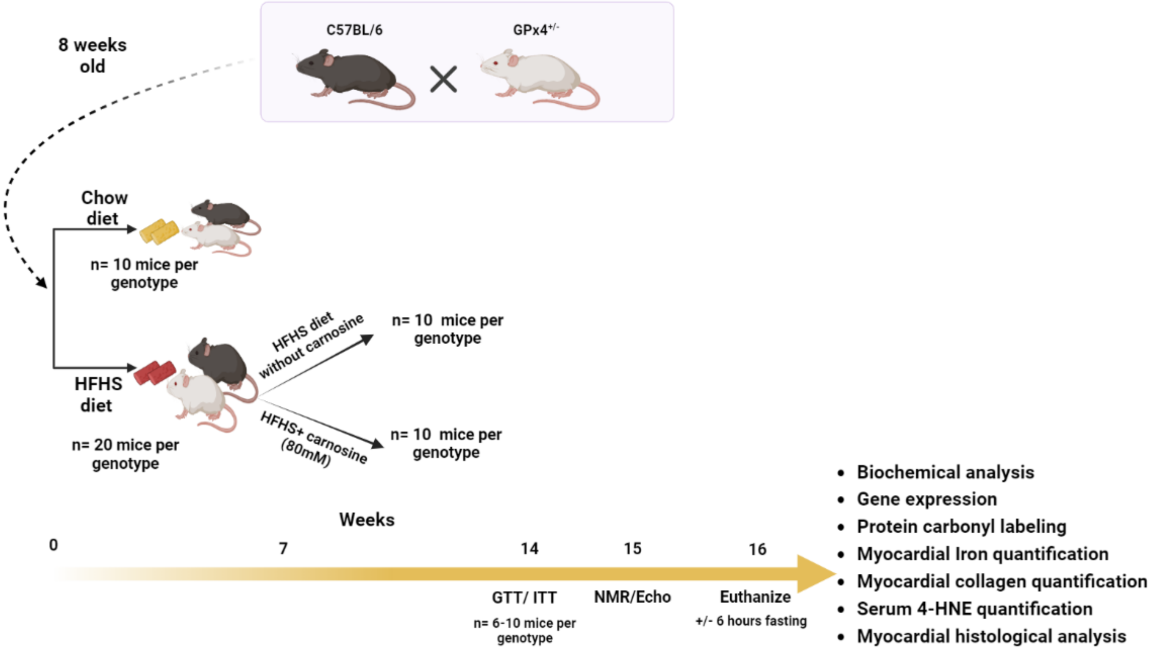

Supplemental Figure 1. Overall study design and endpoints. Adult GPx4$^{+/-}$ and WT mice were crossed to generate pups from both genotypes. At the age of 2 months, male GPx4$^{+/-}$ and WT littermates were randomly assigned to either normal chow diet or HFHS diet. Seven weeks after the diet initiation, half of the HFHS diet group was randomly selected to receive carnosine added to their drinking water at a final concentration of 80 mM. Glucose and insulin tolerance tests (GTT/ITT) were performed at week 14 (7 weeks after onset of carnosine treatment). Echocardiography and whole body NMR were performed one week before the end of the intervention to examine cardiac function and body composition, respectively. At the end of the intervention the mice were euthanized, and the hearts were collected, and flash frozen in liquid nitrogen for further biochemical analysis.

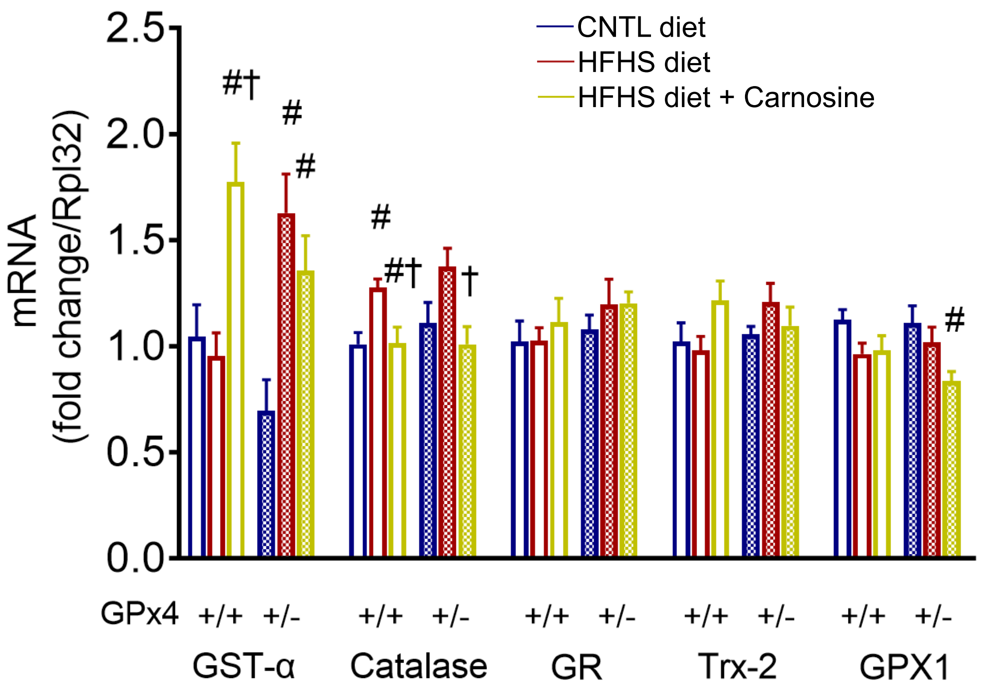

Supplemental Figure 2. Myocardial antioxidant gene expression. Shown are the relative mRNA for each target gene listed on X-axis in both WT ($GPx4^{+/+}$) and $GPx4^{+/-}$ mice. Values are mean ± SEM, using one-way ANOVA with post-hoc Tukey's tests for multiple comparisons within the same genotype: #P < 0.05 vs CNTL, †P<0.05 vs. HFHS. n=4-6 mice per treatment group. +/+, wild type; +/-, GPx4 heterozygote, CNTL, control diet; HFHS, high fat high sucrose diet, GST-α, glutathione S-transferases; GR, glutathione reductase; Trx-2, thioredoxin 2; GPx1, glutathione peroxidase 1.

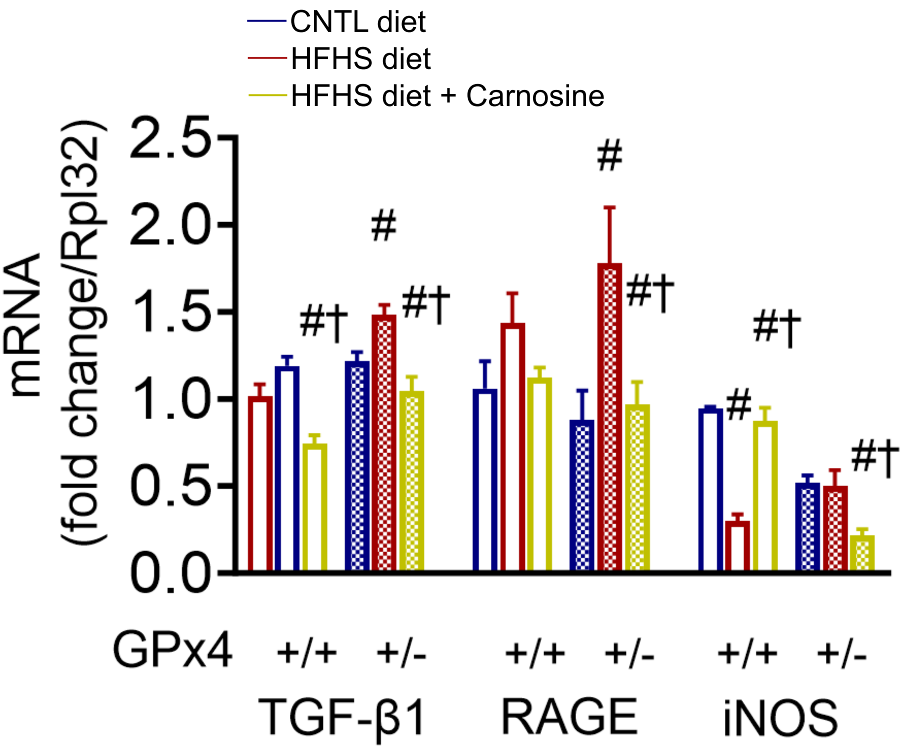

Supplemental Figure 3. Inflammatory gene expression in myocardial tissues. Shown are the relative mRNA for each target gene listed on X-axis in both WT ($GPx4^{+/+}$) and $GPx4^{+/-}$ mice. Values are mean ± SEM, using one-way ANOVA with post-hoc Tukey's tests for multiple comparisons within the same genotype: #P < 0.05 vs CNTL, †P<0.05 vs. HFHS. n=4-6 mice per treatment group. +/+, wild type; +/-, GPx4 heterozygote; CNTL, control diet; HFHS, high fat high sucrose diet, TGF-β, transforming growth factor beta-1; RAGE, receptor for advanced glycation end products; iNOS, inducible nitric oxide.

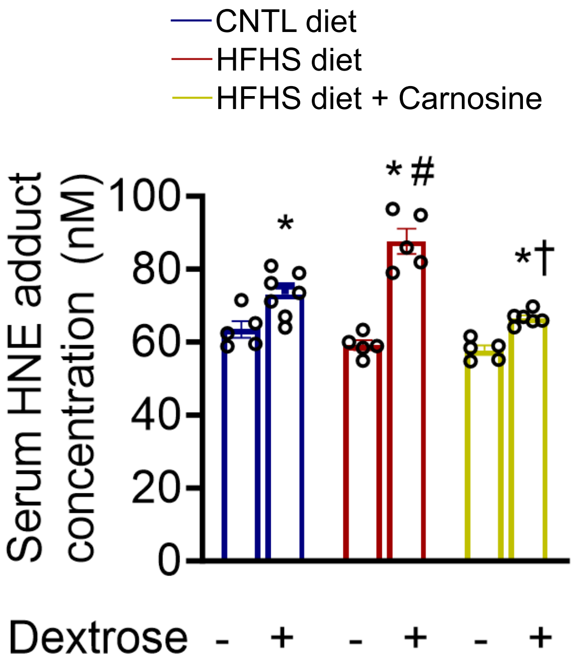

Supplemental Figure 4. **Effect of L-carnosine treatment on 4-HNE protein adducts in serum of WT mice.** Serum 4-HNE protein adduct concentration in WT mice within each intervention group. Values are mean ± SEM with one-way ANOVA with post-hoc Tukey's tests for multiple comparisons within the same genotype: *P<0.05 vs no dextrose; #P<0.05 vs CNTL + dextrose, †P<0.05 vs. HFHS + dextrose. n=4-6 mice per treatment group. CNTL, control diet; HFHS, high fat high sucrose diet.

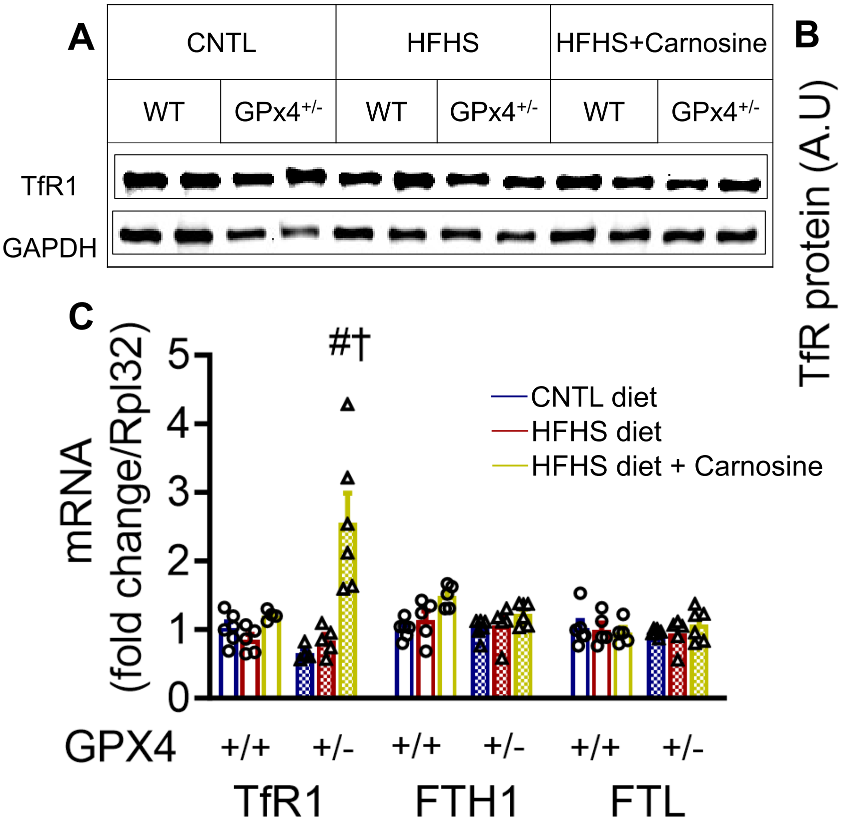

Supplemental Figure 5. **Iron metabolism protein expression in myocardial tissues.** Expression of iron metabolism genes in myocardial tissues from each group. Shown in **(A)** is a representative immunoblot of TfR1 protein, along with densitometry analysis in **(B).** Shown in **(C)** is relative mRNA for each of the iron handling genes listed on X-axis in both WT ($GPx4^{+/+}$) and $GPx4^{+/-}$ mice. Values are mean ± SEM, using one-way ANOVA with post-hoc Tukey's tests for multiple comparisons within the same genotype: #P < 0.05 vs CNTL, †P<0.05 vs. HFHS. n=4-6 mice per treatment group. +/+, wild type (WT); +/-, GPx4 heterozygote; CNTL, control diet; HFHS, high fat high sucrose diet, ns, not significant
