# Supplementary material for: Iron scavenging and suppression of collagen cross-linking underlie antifibrotic effects of carnosine in the heart with obesity
Source: Front Pharmacol. 2024 Jan 3;14:1275388. doi: 10.3389/fphar.2023.1275388 (PMC10859874; doi:10.3389/fphar.2023.1275388)
Supplement: Supplementary file 2 [file DataSheet2.zip › Suppl. Tables.PDF]

Supplementary Table 1. Mass spectrometry analysis of carnosine stability in drinking water

Sample	Base peak (m/z)	Peak area
**Freshly prepared**	227.11	245851019.1 ± 2.6*10^6
**Stored at room temperature for 96 hrs.**	227.11	317850856.2 ± 1.3*10^6

Values are mean ± SEM., n= 3 per sample type. Sample were analyzed using mass spectrum-t-SIM method.

Supplementary Table 2. Primer sequences used with qRT-PCR.

Target	Sense (5’ – 3’)	Antisense (3’ – 5’)	Source/Ref.
**Wild-type GPx4**	CCCCTGCCCTTCTGGACTATTGG	GGCCCTGGTTTCTATGTA	Katunga, 2015
**GPx4+/-**	GTAGGATATGCCCTTGAC	-	KATUNGA, 2015
**GPX4**	TGAGGCAAAACTGACGTAAACTACA	GCTCCTGCCTCCCAAACTG	GOSBELL, 2006
**GST-α1**	CCGTGCTTCACTACTTCAAT	GCATCCATGGGAGGCTTTCT	ZHU 2008
**Catalase**	GACATGGTCTGGGACTTCTG	GTAGGGACAGTTCACAGGTA	ZHU 2008
**GR**	TGCCTGCTCTGGGCCATT	CTCCTCTGAAGAGGTAGGAT	GOSBELL, 2006
**Trx-2**	CAGCCTCTGGCACATTTCCT	GTTCGGCTTCTGGTTTCCTTT	ZHU 2008
**GPX1**	CTCACCCGCTCTTTACCTTCCT	ACACCGGAGACCAAATGATGTAC T	GOSBELL, 2006
**RAGE**	GCATCAGGGTCACAGAAACC	ATTGGGATGGAATGTGGGGG	KATUNGA, 2015
**TGFB1**	ACCCTGCCCCTATATTTGGA	TGGTTGTAGAGGGCAAGGAC	LI, 2012
**iNOS**	CCCTTCCGAAGTTTCTGGCAGCAGC	CCCTTCCGAAGTTTCTGGCAGCA GC	HEO, 2010
**TfR1**	TGGCTGAAACGGAGGAGACAGA	TGGCTCAGCTGCTTGATGGTGT	YU,2020
**FTH1**	AAGATGGGTGCCCCTGAAG	CCAGGGTGTGCTTGTCAAAGA	YU,2020
**FTL**	CGGGCCTCCTACACCTACCT	CCCTCCAGAGCCACGTCAT	YU,2020
